# Anticancer potential of *Reynoutria japonica* Houtt. *in vitro* root extract on lymphoma cell lines

**DOI:** 10.3389/fphar.2026.1904230

**Published:** 2026-08-14

**Authors:** Julia Sroka, Mikołaj Jacyno, Dariusz Nowicki, Wojciech Makowski, Agnieszka Szopa, Paweł Kubica, Aleksandra Królicka, Małgorzata Stasiłojć

**Affiliations:** 1 Laboratory of Biologically Active Compounds, Intercollegiate Faculty of Biotechnology UG and MUG, University of Gdansk, Gdansk, Poland; 2 Department of Immunology, Mossakowski Medical Research Institute, Polish Academy of Sciences, Warsaw, Poland; 3 Doctoral School of Translational Medicine, Mossakowski Medical Research Institute, Polish Academy of Sciences, Centre of Postgraduate Medical Education, Warsaw, Poland; 4 Department of Bacterial Molecular Genetics, Faculty of Biology, University of Gdansk, Gdansk, Poland; 5 Department of Botany, Physiology and Plant Protection, Faculty of Biotechnology and Horticulture, University of Agriculture in Kraków, Kraków, Poland; 6 Department of Medicinal Plant and Mushroom Biotechnology, Jagiellonian University Medical College, Kraków, Poland; 7 Department of Cell Biology and Immunology, Intercollegiate Faculty of Biotechnology UG and MUG, Medical University of Gdansk, Gdansk, Poland

**Keywords:** apoptosis, catechins, Galleria mellonella, Japanese knotweed, lymphoma, secondary metabolites

## Abstract

**Background:**

Plant-derived polyphenols, including catechins, have been extensively studied for their anticancer properties for years. However, complex plant extracts may exhibit enhanced activity compared to isolated compounds owing to synergistic/additive phytochemical interactions. To date, the anticancer potential of extracts from *in vitro*-grown roots of *Reynoutria japonica* Houtt., a plant rich in catechins, has not been investigated.

**Purpose:**

This study aimed to evaluate the anticancer activity of *R. japonica* root extract obtained from *in vitro* culture and to compare its effects with isolated catechins, with particular emphasis on the extract’s potent synergistic/additive effects.

**Methods:**

Methanolic root extracts and pure catechins were tested against five human cancer cell lines (A549, MDA-MB-231, Ramos, Namalwa, Raji) and human fibroblasts (BJ) as a control using the MTT assay. The mode of cell death was assessed by flow cytometry and Annexin V-FITC/PI staining. *In vivo* toxicity of the extract and pure compounds was evaluated in the insect model *Galleria mellonella*.

**Results:**

The root extract induced a reduction in cancer cell viability comparable to that observed for isolated catechins, despite containing substantially lower concentrations of these compounds. This effect was most pronounced in lymphoma cells, which showed the highest sensitivity to extract treatment. Flow cytometric analysis indicated that the extract predominantly triggered apoptotic cell death.

**Conclusion:**

Despite its low catechin concentration, the extract from roots of *R. japonica* Houtt. Exhibits biological activity comparable to that of higher concentrations of pure catechins, suggesting possible synergistic or additive interactions within the phytochemical matrix. Furthermore, toxicological evaluation in the insect model revealed no adverse effects of the extract at biologically active doses.

## Highlights

- Extracts from *in vitro*-cultured *Reynoutria japonica* roots reduce cancer cell viability.
- The extracts behave similarly to pure compounds, despite their lower catechin content.
- Lymphoma cells show the highest sensitivity to the extract.
- The extract and catechins mainly induce apoptosis in cancer cells.
- The active extract doses show no toxicity in a *G*. *mellonella* model.

## Introduction

Regardless of social standing or financial means, cancer is a major health issue in all populations. Cancer is the cause of nearly one in six fatalities (16.8%) and one in four deaths (22.8%) attributed to non-communicable diseases (NCDs) globally (Bray et al., 2024). In 2022, there were around 10 million cancer-related deaths and an estimated 20 million new cases. With 12.4% of all cases, lung cancer is the most frequently diagnosed cancer globally. Prostate, colorectal, and breast cancers are the next most often diagnosed cancers (Bray et al., 2024; Lei et al., 2024). Depending on the type of cancer, impoverished nations may not have easy access to certain preventive measures and treatments. Even industrialised nations face challenges in treating cancer because of the growing resistance to targeted therapies and chemotherapeutic agents (World Health Organization, 2020).

Currently, multidrug resistance (MDR) is becoming a major issue in the fight against cancer. Similar to the concept frequently used in antibiotic treatment, MDR is defined in the context of cancer treatment as the capacity of cancer cells to withstand treatment with a range of anticancer agents. There is mounting evidence that MDR is caused by chemotherapeutic medicines’ enhanced efflux, which decreases cancer cells’ ability to absorb medications (Emran et al., 2022; Zou et al., 2024). Oncogene mutations, tumour microenvironment alterations, tumour heterogeneity, target-site mutations, or epigenetic modifications can all contribute to the development of MDR (Emran et al., 2022).

In the face of this growing issue, we are continually searching for new solutions and alternatives to existing therapies, and the world of plants offers promising prospects. Many plant-derived bioactive secondary metabolites are now used both directly as medicines or supplements and as raw materials for semi-synthetic modifications (Wawrosch and Zotchev, 2021). Plants rich in substances with medicinal properties have been used in folk medicine for thousands of years. It is increasingly common to revisit sources and search for health-promoting plant species with promising pharmacological potential. This knowledge, along with the potential use of yet unexplored species, is of great value for the discovery of new bioactive natural products (Wawrosch and Zotchev, 2021). Among the numerous classes of secondary plant metabolites, polyphenols can inhibit multiple pathways of carcinogenesis, including cell transformation, invasion, metastasis, and angiogenesis. This is possible through their activation of kinases, as well as by reducing the pool of transcription factors, controlling the cell cycle, and inducing apoptotic cell death (Lyu et al., 2020; Tufail et al., 2025).

The catechin (flavan-3-ol) belongs to the subgroup of polyphenols, which are found in many plant species, while its very rich source is *R. japonica* Houtt (Japanese knotweed), which is the subject of this study. In addition to catechin, *R. japonica* has the ability to synthesise and accumulate (−)-epigallocatechin gallate (EGCG) (−)-epicatechin gallate (ECG) (−)-epigallocatechin (EGC), and (−)-epicatechin (EC) (Makowski et al., 2024). They are representatives of metabolites with health-promoting effects (Farhan, 2022; Steinmann et al., 2013). They belong to the antioxidant group and play a key role in protection against oxidative stress, which can lead to DNA damage, mutations, and genomic instability, thereby contributing to cancer initiation (Sweety et al., 2025). This effect can be attributed to the presence of oxidation-sensitive phenolic groups in their structure (Lambert and Elias, 2010). In addition, catechin has been shown to induce apoptosis in cancer cells by activating caspases-3 and -9, leading to cell cycle arrest in the G1 phase. EGCG also promotes cancer cell death by modulating proteins that regulate apoptosis. All this leads to inhibition of proliferation and promotes elimination of potentially cancerous cells (Luo et al., 2024). Another important aspect of catechins is their ability to inhibit receptor tyrosine kinase (RTK) pathways, which play key roles in regulating proliferation, cell survival, and angiogenesis. For example, EGCG effectively blocks the activity of receptors such as EGFR (epithelial growth factor receptor), HER2 (Human Epidermal Growth Factor Receptor 2), IGF-1R (Insulin-like Growth Factor 1 Receptor), and VEGFR (Vascular Endothelial Growth Factor Receptor), as well as their intracellular signalling pathways, PI3K/Akt (Phosphoinositide 3-kinase/Protein Kinase B) and MAPK/ERK (Mitogen-Activated Protein Kinase/Extracellular Signal-Regulated Kinase). The effect is to inhibit the expression of tumour-promoting genes, limit tumour growth, and reduce the formation of new blood vessels (Li et al., 2022).

The structural complexity of desirable health-promoting compounds makes them difficult to synthesise both chemically and cost-effectively. For this reason, efforts are being made to develop new guided therapies and assisted treatments, and to design new drugs and supplements based on plant metabolites. As a result, there is growing interest in alternative medicine. Many studies show that the use of natural compounds, such as phytochemicals, is a promising strategy for treating many diseases (Wawrosch and Zotchev, 2021).

*Reynoutria japonica* (syn. *Polygonum cuspidatum/Fallopia japonica*) is a species of the Polygonaceae family, native to southern Asia. Its native range includes Japan, Korea, Taiwan, and northern China, where it occurs in wet, humid, open mountainous areas, as well as on roadsides and the banks of ditches and canals. This plant is also found in North America (Canada and the United States), New Zealand, Australia and Europe (Tokarska-Guzik, 2005). The rhizome and root are key organs of this plant, playing important roles in acclimatisation and adaptation, enabling it to spread and survive unfavourable conditions (Tokarska-Guzik, 2005). They also serve as a reservoir of nutrients and accumulate secondary metabolism products. The substances abundant in Japanese knotweed rhizomes and roots are phenolic compounds–including high levels of catechin and its derivatives (Makowski et al., 2024). These groups of compounds have health-promoting properties, which is why *R. japonica* has been used medicinally in Asia for years and is still listed in the Chinese and European Pharmacopoeia. In traditional Chinese medicine, extracts from the roots of *R. japonica* have been used for decades as analgesics, anti-inflammatory agents, antipyretics, antibacterial agents, and expectorants. They have been used to treat numerous conditions, including asthma, hypertension, heart and circulatory system diseases, inflammation, bacterial, fungal, and viral infections, and cancer (Peng et al., 2013). It should be noted that this species is highly dangerous to the ecosystems in which it lives due to its potential for expansion. *Reynoutria japonica* has developed a range of adaptive and invasive strategies, allowing it to colonize an increasing number of ecological niches and displace native species from them, thereby earning a notorious reputation (Li et al., 2022). It exhibits a high tolerance for environmental pollution and thrives on urban and industrial wastelands, along roads, watercourses, and near mines and railroad embankments. It grows in various types of soil, even colonizing volcanic soils. Its presence has been recorded in soils with a pH range from 3 to 8.5, as well as in saline, polluted, or heavy-metal-contaminated soils (Li et al., 2022). However, as demonstrated in our previous studies (Makowski et al., 2024), it is a source of numerous secondary metabolites, particularly catechin and its derivatives, which are increasingly important in the pharmaceutical industry. We are constantly searching for new drugs and supplements, and trends indicate growing interest in plant-derived natural substances. For this reason, this species, obtained through *in vitro* cultivation independent of environmental conditions, can serve as a good model for biotechnological research aimed at producing plant secondary metabolites for medicinal purposes. In addition, cultivation of this species *in vitro* is quick and simple, and the material obtained is uniform and sterile.

The aim of this study was: (i) to investigate, for the first time, the anticancer activity of extracts from *R. japonica* roots cultivated *in vitro*, (ii) to evaluate the anticancer activity of pure compounds abundant in extracts from *R. japonica* roots, and (iii) to investigate the toxicity of both reference compounds and plant extracts using the *Galleria mellonella* insect model. The study hypothesised that secondary metabolites in *R. japonica* roots exhibit anticancer activity and that synergistic or additive interactions occur among them.

## Materials and methods

### *In vitro* plant cultivation

The study used the anatomical roots of *R*. *japonica*, obtained from two-leaf *in vitro* plants acquired through our previous research (Makowski et al., 2024) (from the collection of the Laboratory of Biologically Active Compounds, University of Gdansk). *Reynoutria japonica* plants were sub-cultured in 1,000 mL jars (eight explants per jar) using 140 mL of ½ Murashige and Skoog (½ MS) medium, solidified with 0.75% agar, containing 3% sucrose, 0.1% 2-(N-morpholino)ethanesulfonic acid (MES), 150 mg/L ascorbic acid, and pH 5.8. Plants were cultivated at 21 °C ± 2 °C under white fluorescence light with a PPFD (Photosynthetic Photon Flux Density) of 100 μmol × m^-2^ × s^-1^ and a photoperiod of 16 h/8 h (light/dark cycle). Plants were collected for further examination after 8 weeks of culture. After harvesting, the roots were separated from the stems and leaves, homogenised, and then freeze-dried for 24 h.

### Preparation of plant extracts and pure tested compounds

Plant extract was prepared following the method of Makowski et al. (2024). Briefly, 100 mg of homogenised, lyophilised root tissue powder was extracted with 6 mL of 80% HPLC-grade methanol in an ultrasonic bath (Ulsonix Proclean 3.0 DSP, Germany) for 30 min at 21 °C ± 2 °C. Extracts were centrifuged at 425 *g* and evaporated in a vacuum concentrator (CentriVap Concentrator, Labconco, United States). For cell experiments, the pellet obtained after evaporation was resuspended in culture medium and filtered through 0.22 μm syringe filters (Milex®GP, Millipore, Germany).

Reference polyphenols (−)-epigallocatechin gallate (EGCG, Pol-Aura, Poland) (−)-epicatechin gallate (ECG, Pol-Aura, Poland) (−)-epigallocatechin (EGC, Roth, Germany), and (−)-epicatechin (EC, Roth, Germany) were dissolved in pure HPLC-grade methanol to a concentration of 4 mg/mL. Appropriate volumes of catechin solutions were transferred to sterile tubes, evaporated under a laminar flow hood, and the resulting pellets were resuspended in culture media (DMEM, EMEM, RPMI 1640 from DIAG-MED, Poland).

For *in vivo* toxicity assessment in *G*. *mellonella*, stock solutions of *R. japonica* extract and reference polyphenolic compounds (EC, ECG, EGC, EGCG) were prepared in a sterile vehicle at concentrations corresponding to the highest concentrations of the plant extract and the pure substances used to analyse cell viability (mg/kg of caterpillar). The appropriate amounts of root extract and catechin slopes were evaporated, then suspended in ethanol. The samples were topped up with NaCl (0.9%) solution. The final ethanol concentration in the prepared injection solutions was 10%.

### Cell lines and cell cultures

The cell lines normal human fibroblasts (BJ), non-small cell lung cancer (A549), triple-negative breast cancer (MDA-MB-231), and Burkitt’s lymphoma (Ramos, Namalwa, Raji) were obtained from ATCC (American Type Culture Collection, United States). BJ cells were cultured in EMEM (Eagle’s Minimum Essential Medium, Corning, United States), A549 and MDA-MB-231 cells in DMEM (Dulbecco’s Modified Eagle Medium, Corning, United States), and Ramos, Namalwa, and Raji cells in RPMI 1640 medium (Roswell Park Memorial Institute 1,640, Corning United States), each supplemented with 10% foetal bovine serum (FBS, ATCC, United States). All cultures were maintained at 37 °C in a humidified atmosphere with 5% CO_2_.

### Cytotoxicity assay

To assess the viability of cells treated with root extracts or pure compounds, the MTT (3-(4,5-Dimethylthiazol-2-yl)-2,5-Diphenyltetrazolium Bromide, Merck, Germany) assay was performed. Cells were seeded in 96-well plates at a density of 1 × 10^4^ cells per well for BJ and A549, and 2.5 × 10^4^ cells per well for MDA-MB-231, then cultured overnight to allow attachment. Subsequently, the cells were exposed to varying concentrations of pure compounds (0–80 µM) or root extracts (0–1,000 µg dry weight (dw)/mL) for 24 or 48 h. Following incubation, 12 µL of 50 µM MTT solution in PBS was added to each well, and the cells were incubated for an additional 2 h. The medium was then removed, and cells were washed with PBS. Finally, DMSO was added to dissolve the cells and formazan crystals, and absorbance was measured at 570 nm using a Synergy H1 Microplate Reader (Agilent Technologies, United States).

For the Ramos, Namalwa, and Raji cell lines, cells were seeded in 96-well plates at a density of 5 × 10^4^ cells per well. Pure compounds or root extracts were applied at concentrations ranging from 0 to 80 µM or 0 to 1,000 µg dw/mL, respectively. After 24 or 48 h of incubation, 12 µL of 50 µM MTT solution in PBS was added to each well, followed by an additional 2 h incubation. Subsequently, an equal volume of isopropanol containing 0.04 M HCl was added to dissolve both the cells and the formazan crystals. Absorbance was measured at 570 nm using a Synergy H1 Microplate Reader (Agilent Technologies, United States).

### Cell cycle analysis

To prepare A549, BJ, and MDA-MB-231 cell lines for cell cycle analysis, 5 × 10^4^ cells were seeded in 24-well plates and incubated overnight at 37 °C in a humidified atmosphere with 5% CO_2_. Following incubation, cells were treated with catechins at a concentration of 80 μM or plant extracts at 1,000 µg dw/mL for 48 h. Untreated cells served as the negative control. After incubation, the medium containing dead cells was transferred to adjacent wells, and the viable cells were detached using trypsin. The cells were then suspended in the previously transferred medium. Subsequently, cells were centrifuged and washed twice with PBS. The cell pellet was resuspended in ice-cold 70% ethanol, incubated on ice for 15 min, and stored at −20 °C.

For Ramos, Namalwa, and Raji cell lines, 1 × 10^6^ cells were seeded in 24-well plates and treated with catechins (80 μM) or plant extracts (1,000 µg dw/mL) for 48 h (catechins and extracts). Untreated cells served as a negative control. After incubation, cells were centrifuged and washed twice with PBS. The cell pellet was resuspended in ice-cold 70% ethanol, incubated on ice for 15 min, and stored at −20 °C.

Fixed cells were centrifuged again and resuspended in 200 µL of staining buffer containing PBS, 2.5 μg/mL DAPI (Merck, Germany), and 0.5 mg/mL RNase (EURx, Poland). Data were acquired using a CytoFLEX flow cytometer (Beckman Coulter, United States) with excitation at 358 nm and emission detected at 461 nm. Ten thousand events were taken for each sample, in three technical repetitions, and subsequently analysed using Kaluza software version 2.1 (Beckman Coulter, United States) with the cell cycle analysis module.

### Detection of apoptosis using annexin V-fitc/PI staining

Cell death type was assessed using the BD Pharmingen™ FITC Annexin V Apoptosis Detection Kit I (BD, United States), following the manufacturer’s instructions. Cells were prepared as described previously for cell cycle analysis, with the modification that after two washes with PBS, the cells were resuspended in 100 µL of binding buffer. Annexin V-FITC (5 µL) and propidium iodide (PI) (5 µL) were added, and the samples were incubated for 15 min in the dark at room temperature. The reaction was then stopped by adding additional 400 µL of binding buffer. Samples were analysed by flow cytometry (CytoFLEX, BD, United States) using a blue laser (λ_ex = 488 nm) and fluorescence detection at 525/40 nm for Annexin V-FITC and 585/42 nm for PI.

### *In vivo* toxicity assessment in *G. mellonella*

Larvae of *G. mellonella* were obtained from a commercial livestock supplier (Egzotic-room, Poland) and stored in the dark at 15 °C prior to use (Karczewska et al., 2024). Only healthy, cream-coloured larvae weighing 300–350 mg, without signs of melanisation or physical damage, were selected. Larvae were randomly assigned to treatment groups (n = 10 per group) and placed in sterile Petri dishes. Prior to injection, each larva was surface-sterilised by gently swabbing with 70% ethanol. Working solutions were freshly prepared immediately before use. A Hamilton microsyringe (10 µL) was disinfected in 70% ethanol and rinsed twice with sterile PBS before each series of injections. Each larva received 10 µL of the test solution (*R. japonica* extract and reference polyphenolic compounds whose preparation procedure is described above) injected into the hemocoel *via* the last left proleg. The control group received the same volume of sterile physiological saline. Following injection, larvae were incubated in the dark at 37 °C. Survival was monitored at 24 h intervals for 120 h. Mortality was recorded when larvae showed complete melanisation and/or failed to respond to gentle tactile stimulation, including inability to right themselves when placed on the dorsal side (Budziaszek et al., 2023). The use of *G. mellonella* larvae is consistent with the 3 R principle, as this invertebrate model provides a preliminary *in vivo* screening platform that can reduce the number of vertebrate animals required at early stages of biological and toxicological evaluation. According to applicable regulations, experiments using *G. mellonella* larvae do not require approval from an institutional animal ethics committee, as this species is not covered by legislation governing the use of protected vertebrate animals in experimental procedures.

### Quantitative and qualitative analysis of extracts–high-performance liquid chromatography

Phenolic compounds in extracts were quantified using HPLC-DAD according to Makowski et al. (Makowski et al., 2024). Analyses were performed on a Merck-Hitachi LaChrom Elite system equipped with an L-2455 diode-array detector and a Purospher RP-18e column (250 × 4 mm, 5 μm; Merck, Germany). Ethanolic extracts prepared as described above were filtered through 0.22 μm PTFE filters and injected directly. The results of the analysis are included in the Supplementary Table S1.

### Statistical analysis

All cell line experiments were performed in three biological replicates, each conducted in triplicate. Data are presented as mean ± standard deviation (SD) of the three biological replicates. Statistical significance was considered at *p* ≤ 0.05, *p* ≤ 0.01, *p* ≤ 0.001, and *p* ≤ 0.0001. Student’s t-test or Welch’s *t*-test (depending on the homogeneity of variances) with Holm–Bonferroni correction was used for pairwise comparisons between samples. Statistical analysis were performed using MS Excel (Microsoft, United States).

For *in vivo* toxicity assessment in *G. mellonella*, results were analysed using Kaplan–Meier survival curves, and statistical differences between groups were evaluated with the log-rank (Mantel–Cox) test. A *p*-value <0.05 was considered statistically significant. Statistical analysis was performed using GraphPad Prism (GraphPad Software, United States).

## Results

**FIGURE 1:**
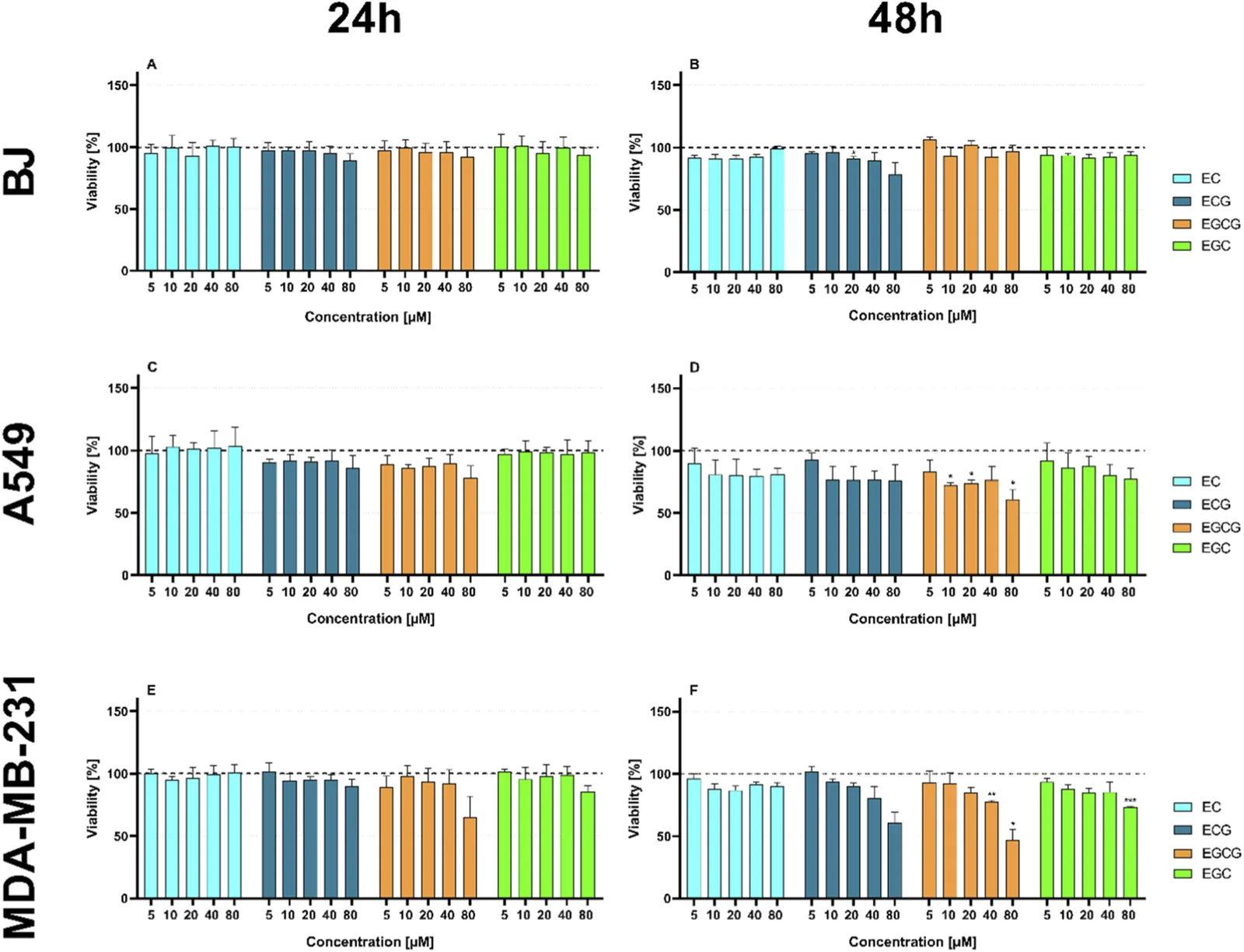
Influence of catechins (EC, ECG, EGCG, EGC) on the viability of BJ **(A,B)**, A549 **(C,D)**, and MDA-MB-231 **(E,F)** cells. Cells were treated with different concentrations of catechins for 24 **(A, C**, **E)** or 48 h **(B, D,F)**. Viability was measured by the MTT assay, and all results were normalised to the non-treated negative control (dashed line). Data are shown as mean ± SD from three biological experiments. *p < 0.05, **p < 0.01, ***p < 0.001 vs. non-treated control.

The effects of four catechins–EC, ECG, EGCG, and EGC–on normal human fibroblasts (BJ) and two tumour cell lines (A549, MDA-MB-231) were evaluated using the MTT assay (Figure 1). Cells were treated with varying concentrations of the compounds for 24 (Figures 1A,C,E) or 48 h (Figures 1B,D,F). None of the tested catechins reduced the viability of BJ cells (Figures 1A,B). In contrast, EGCG significantly decreased the number of viable A549 pulmonary tumour cells after 48 h when compared to the untreated cells (Figure 1D). A similar effect was observed in the triple-negative breast cancer cell line MDA-MB-231, where both EGCG and EGC induced a significant decrease in the number of viable cells after 48 h (Figure 1F). Since catechins can reduce MTT to formazan independently of cellular metabolism, they can produce a signal unrelated to cell viability, making the assay inherently unreliable for suspension cells. Consequently, MTT results for Ramos, Namalwa, and Raji cells are not presented here.

**FIGURE 2:**
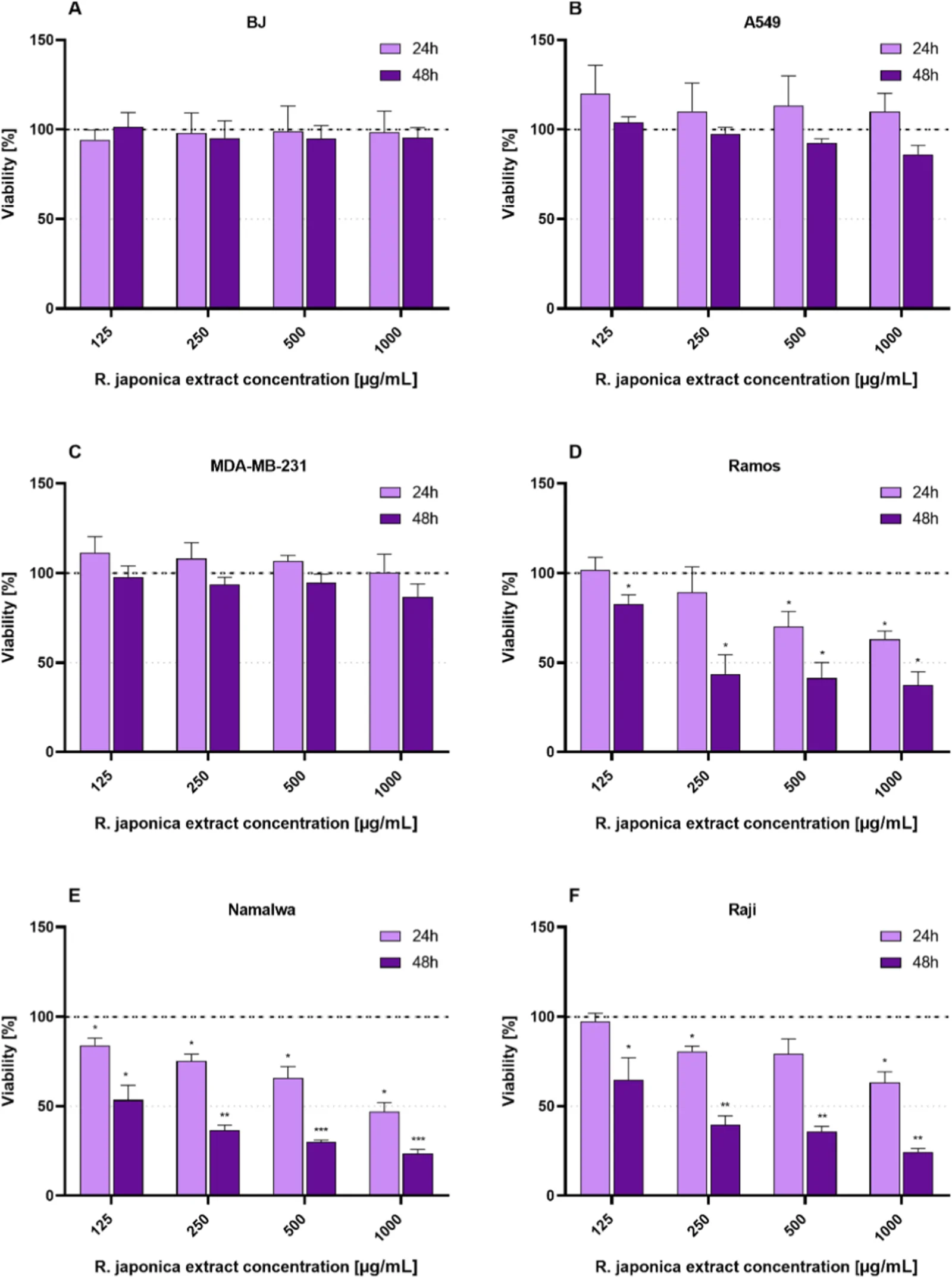
Influence of *Reynoutria japonica* root extract on the viability of BJ **(A)**, A549 **(B)**, MDA-MB-231 **(C)**, Ramos **(D)**, Namalwa **(E)**, and Raji **(F)** cells. Cells were treated with different concentrations of catechins for 24 or 48 h. Viability was measured by the MTT assay, and all results were normalised to the non-treated negative control (dashed line). Data are shown as mean ± SD from three biological experiments. *p < 0.05, **p < 0.01, ***p < 0.001 vs. non-treated control.

We next evaluated the effect of *R. japonica* root extract on the viability of six tested cell lines, including B-cell lymphoma Ramos, Namalwa, and Raji cell lines (Figure 2). The methanolic extract, equivalent to 1,000 µg of dry root weight, was evaporated, and the residue resuspended in culture medium to a final concentration of 1,000 µg dw/mL. A significant reduction in the number of viable cells was observed exclusively in the lymphoma cell lines Ramos, Namalwa, and Raji after 24 h of treatment, with this effect becoming more pronounced at 48 h (Figures 2D–F). No statistically significant differences were observed for the BJ control (Figure 2A), A549 (Figure 2B), and MDA-MB-231 (Figure 2C).

**FIGURE 3:**
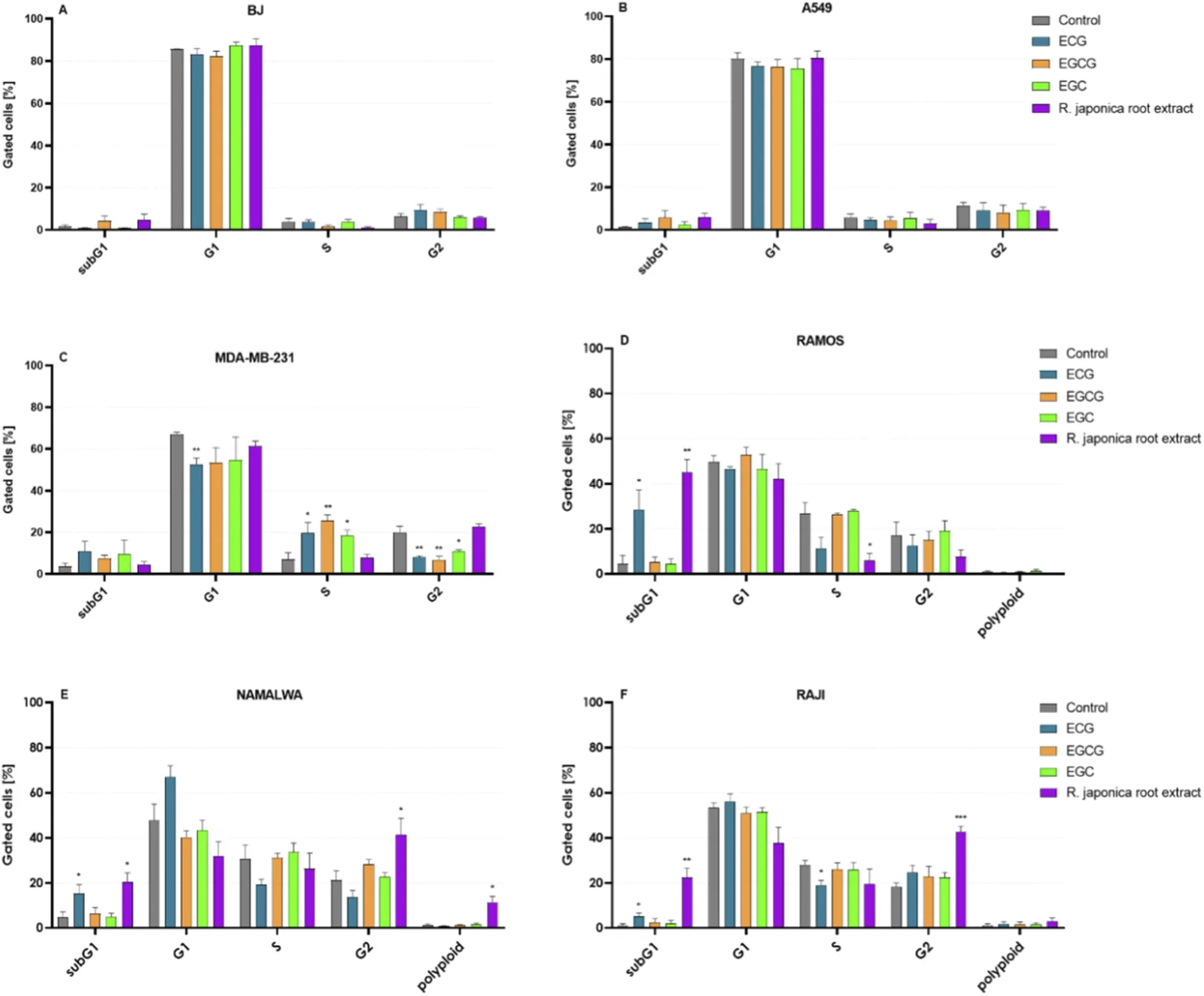
Influence of catechins (ECG, EGCG, EGC) and *Reynoutria japonica* root extract on the cell cycle of BJ **(A)**, A549 **(B)**, MDA-MB-231 **(C)**, Ramos **(D)**, Namalwa **(E)**, and Raji **(F)** cells. Cells were treated with catechins at a concentration of 80 µM or with root extract at a concentration of 1,000 µg dw/mL for 48 h. Data are shown as mean ± SD from three biological experiments. *p < 0.05, **p < 0.01, ***p < 0.001 vs. non-treated control.

To further investigate the effects of catechins and root extract on cell condition, we analysed the cell cycle of all tested cell lines (Figure 3). Cells were treated with catechins at a concentration of 80 µM and root extract at 1,000 µg dw/mL for 48 h. The concentration was selected on the basis of the highest tested concentrations in the MTT assays, which showed the most pronounced effects (Figure 1 and Figure 2). Since EC did not affect cell viability in the MTT assay, it was excluded from this experiment. No statistically significant differences in cell cycle were observed after treatment of BJ and A549 cells with the compounds studied (Figures 3A,B). Despite minimal effects of catechins on MDA-MB-231 viability, significant changes in the cell cycle were observed after treatment with ECG, EGCG, and EGC compared to untreated control cells (Figure 3C). The increased proportion of cells in the S phase, along with a decreased proportion in the G2 phase, suggests S-phase arrest in this cell line. Treatment with the root extract induced a significant accumulation of cells in the subG1 phase in Ramos, Namalwa, and Raji lymphoma cell lines (Figures 3D–F), indicative of apoptosis. In Ramos cells, proliferation was markedly inhibited, as evidenced by reduced proportions of cells in the S and G2 phases (Figure 3D). In Namalwa and Raji cells, the extract additionally caused a significant increase in the G2 phase population (Figures 3E,F). Interestingly, all lymphoma cell lines exhibited a small fraction of polyploid cells, which was not observed in adherent cells. While the percentage of polyploid cells remained very low in the Ramos and Raji cell lines, it increased significantly in Namalwa cells following treatment with the root extract (Figure 3E).

**FIGURE 4:**
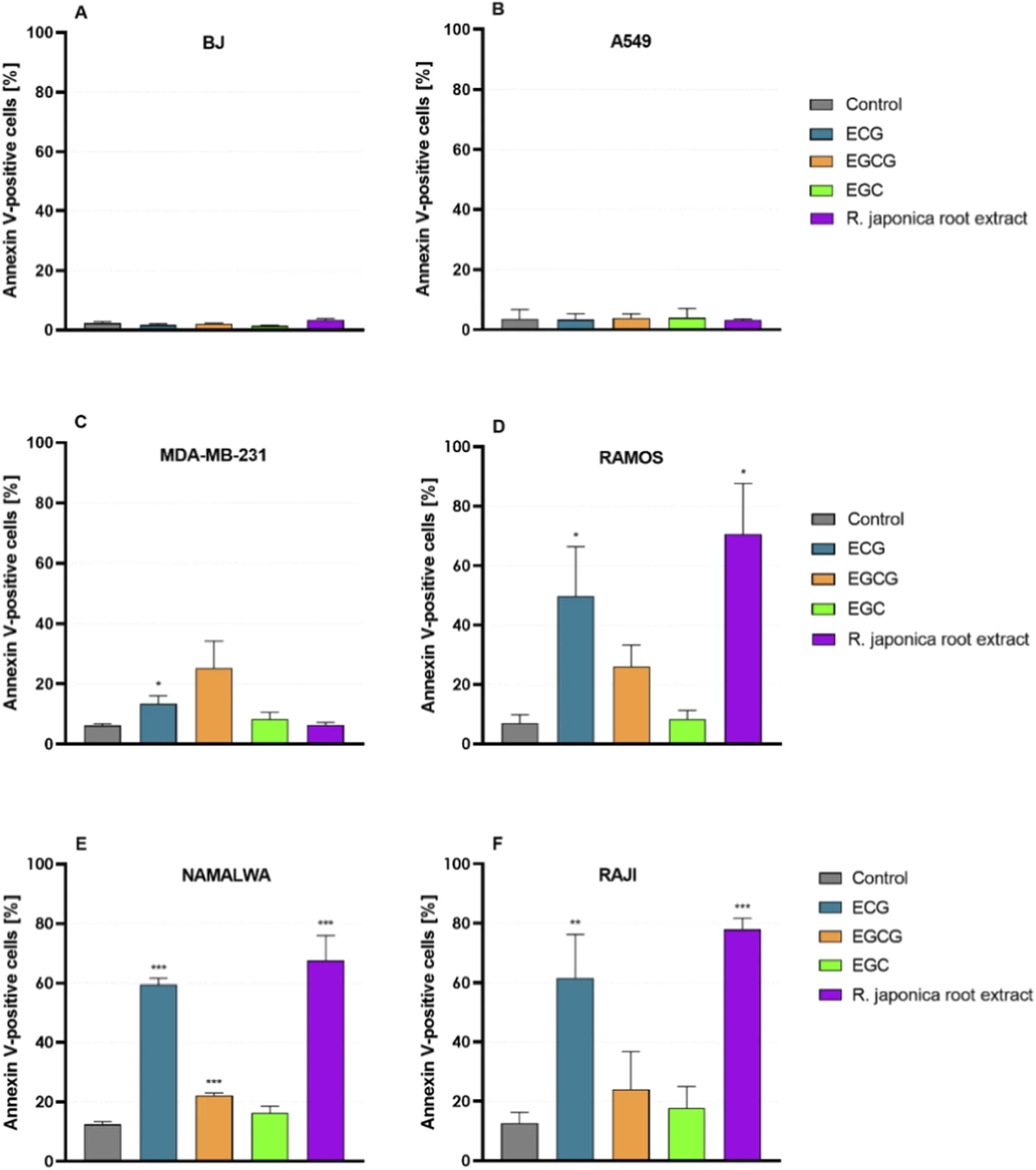
Induction of apoptosis by catechins (ECG, EGCG, EGC) and *Reynoutria japonica* root extract in BJ **(A)**, A549 **(B)**, MDA-MB-231 **(C)**, Ramos **(D)**, Namalwa **(E)**, and Raji **(F)** cells as determined by Annexin V apoptosis assay. Cells were treated with catechins at a concentration of 80 µM or with root extract at a concentration of 1,000 µg dw/mL for 48 h. Data are shown as mean ± SD from three biological experiments. *p < 0.05 vs. non-treated control.

To assess whether catechins or *R. japonica* root extract induce apoptosis, cells were subjected to double staining with Annexin V and PI to differentiate live, apoptotic, and necrotic populations (Figure 4). Treatments were applied at concentrations of 80 µM for catechins and 1,000 µg dw/mL for root extract over 48 h. Cells positive for Annexin V were classified as apoptotic, including those in late apoptosis. A slight but statistically significant increase in apoptotic MDA-MB-231 cells was observed following treatment with ECG (Figure 4C), whereas EGCG treatment showed a similar trend (though not statistically significant). For A549 cells (Figure 4B) we observe a result similar to the BJ control (Figure 4A). In Ramos cells, both ECG and EGCG induced apoptosis, although the effect of the latter was not statistically significant (Figure 4D). In the Namalwa and Raji cell lines, treatment with pure ECG yielded a statistically significant result (Figures 4E,F). The most pronounced induction of apoptosis was detected in Ramos, Namalwa, and Raji cells treated with *R. japonica* root extract. None of the compounds tested induced necrosis–the levels of necrotic cells were not significantly different from those in the non-treated control (data not shown).

**FIGURE 5:**
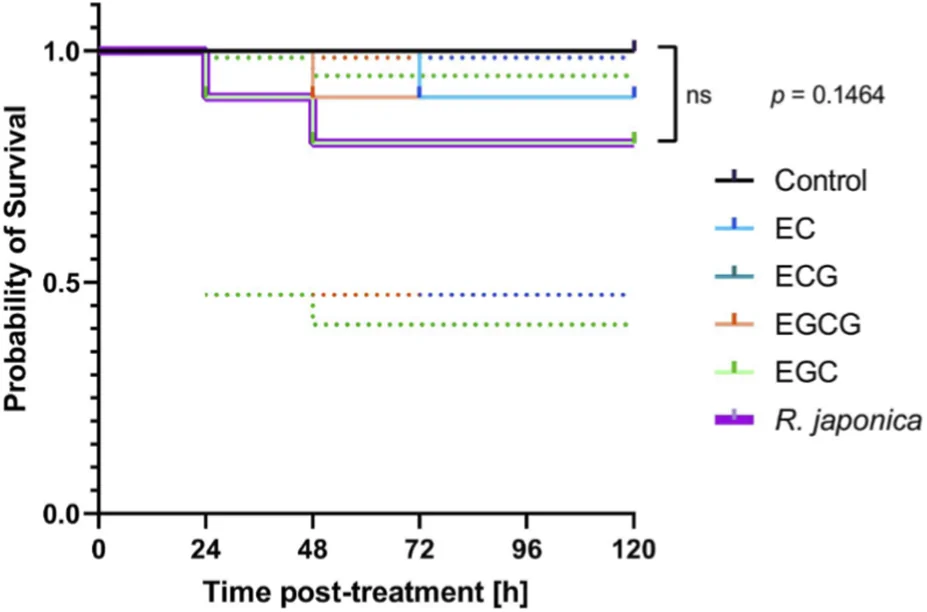
Survival curves of *Galleria mellonella* larvae following treatment with *Reynoutria japonica* extract (violet line), its reference polyphenolic constituents: epicatechin (EC, light blue line), epicatechin gallate (ECG, dark blue line), epigallocatechin gallate (EGCG, orange line), epigallocatechin (EGC, green line), or saline 0.9% used as a negative control. Larvae were injected with test compounds and monitored for 120 h post-treatment. No statistically significant differences in survival probability were observed between *Reynoutria japonica* extract treatment and saline-treated control (Mantel–Cox log-rank test, *p* = 0.1464).

Subsequently, to assess the toxicity of the extract from *R. japonica* roots and the reference compounds, a toxicity test was conducted using a simple insect model. The analysis of moth larvae survival after administration of various extracts is presented in Kaplan–Meier curves (Figure 5). In the control group, the percentage of surviving individuals remained high throughout the observation period (up to 120 h). The application of selected catechins (EGC, EGCG) and *R. japonica* extract resulted in a decrease in larval survival, especially in the first 48 h of the experiment. A moderate decrease in survival was also observed in the EC and ECG groups, but the curves indicate a relatively mild effect.

Despite the observed trends, statistical analysis showed no significant differences between the groups (*p* = 0.1464). This means that the observed decrease in survival may have been due to random fluctuations rather than the clear action of the compounds used.

**FIGURE 6:**
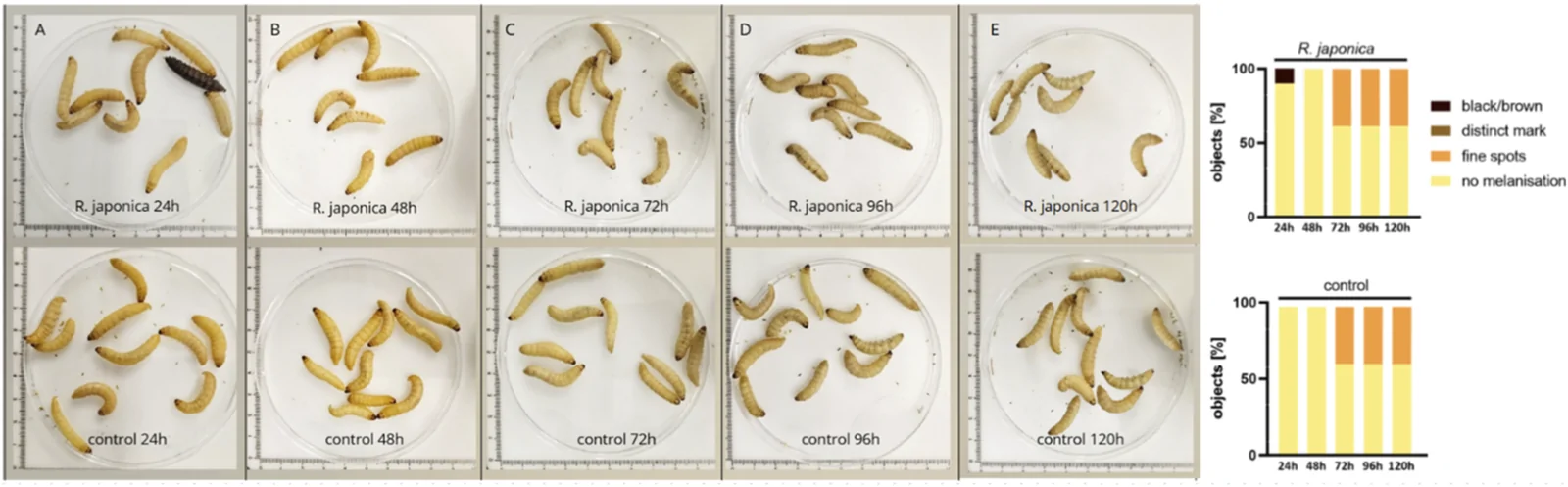
Survival of *Galleria mellonella* larvae following treatment with *Reynoutria japonica* extract and 0.9% saline used as a negative control. **(A)** after 24 h, **(B)** after 48 h, **(C)** after 72 h, **(D)** after 96 h, **(E)** after 120 h.

Additionally, changes in the larvae during the observation period were documented photographically (Figures 6A–E). After applying extracts from *R. japonica* roots, a decrease in viability was observed within the first 48 h. Dead larvae were melanised, turning dark brown, and did not respond to gentle touch.

## Discussion

Due to the growing interest in alternative medicine, numerous studies are currently being conducted to assess the pharmacological potential of natural raw materials obtained from plants and their potential use in the pharmaceutical industry. Systematising and expanding knowledge in this area is of great value for the discovery of new bioactive natural products (Wawrosch and Zotchev, 2021). To efficiently manage the health-promoting benefits of these plants, it is necessary to optimise cultivation methods to ensure access to standardised material rich in desirable bioactive compounds (Ruta et al., 2020). This study aimed to assess the anticancer potential of an *R. japonica* root extract derived from *in vitro* culture and to compare its activity with that of isolated catechins, with a special focus on the extract’s potency and potential synergistic or additive interactions. Additionally, the toxicity of the tested extracts was evaluated using a *G. mellonella* insect model. The *G. mellonella* model provides a useful surrogate *in vivo* system due to its innate immune response partially resembles mammalian host defence (Budziaszek et al., 2023; Gallorini et al., 2024). In particular, larvae several types of haemocytes involved in cellular immunity and activate humoral antimicrobial mechanisms, including antimicrobial peptide production and phenoloxidase-dependent melanisation. Therefore, although *G. mellonella* does not fully reproduce mammalian physiology, it is suitable for preliminary assessment of toxicity and host-associated biological effects of tested compounds.

The dried underground parts of Japanese knotweed are listed as a medicinal raw material in both the Chinese and European Pharmacopoeias. Studies to date clearly indicate the strong anticancer properties of extracts obtained from the roots of *R*. *japonica*. Khuda et al. (2022) demonstrated that this extract effectively inhibits the proliferation of A549, MDA-MB-231, and Hep-G2 cancer cell lines. Similar results were obtained by Kim’s team (Kim et al., 2019), who observed that *R. japonica* radix extract inhibits the proliferation and invasion of hepatocellular carcinoma cells by modulating the MAPK and PI3K/Akt signalling pathways. It has been shown that MAPK promotes gene expression driving cell cycle progression (Santarpia et al., 2012). Moreover, its overactivation leads to uncontrolled cell proliferation (Yamamoto et al., 2006). PI3K/Akt enhances cell survival by inhibiting apoptosis (Mundi et al., 2016). In turn, Wang et al. (2023) demonstrated that the synergistic use of *R. japonica* and *Citri Reticulatae Pericarpium* extracts leads to the inhibition of proliferation, migration, and invasion of breast cancer cells and reduces metastasis in an animal model. Despite numerous reports confirming the anticancer activity of *R. japonica* rhizome extracts obtained from conventionally grown plants, there is currently no data on the activity of such extracts derived from plant material obtained under *in vitro* culture conditions. Cultivation *in vitro* allows for the standardisation of the plant material obtained. Also, it ensures that the culture is free of harmful, toxic environmental factors, such as heavy metals that *R. japonica* accumulates (Makowski et al., 2024).

In our study, we first focused on the cytotoxic effects of pure catechins on both fibroblast cells and five cancer cell lines (Figure 1). Polyphenols, including flavan-3-ols (catechin) could inhibit various pathways of carcinogenesis, including cellular transformation, invasion, metastasis, and angiogenesis. This is possible due to their ability to activate kinases, reduce the pool of transcription factors, regulate the cell cycle, and induce apoptotic cell death (Lyu et al., 2020). For instance, EGCG chemopreventive activity has been demonstrated through modulation of various cellular signalling pathways, including the regulation of proliferation and apoptosis. Moreover, it exerts an inhibitory effect on the initiation, promotion, and progression stages of carcinogenesis. Similar to catechins, EGCG is a well-known antioxidant, capturing the majority of free radicals–such as reactive oxygen species (ROS) and reactive nitrogen species (RNS) – through the xanthine oxidase (Bimonte et al., 2017). None of the tested catechins reduced the number of viable BJ cells (Figures 1A,B). According to the literature, the four catechins exhibit distinct cytotoxic effects on normal human fibroblasts, with EGCG and ECG more toxic than EGC and EC. The evidence comes from multiple studies using MTT and similar viability assays on various normal human fibroblast cell lines. For EGCG, studies have reported IC_50_/LC_50_ values ranging from 78 to 120 μM in normal fibroblasts (Huang et al., 2017; Meng et al., 2008). The MTT assay is a well-established and widely applied method for evaluating cell viability *in vitro*. Due to its sensitivity and suitability for high-throughput screening, the MTT assay is commonly used to evaluate the cytotoxic effects of bioactive compounds (Van Meerloo et al., 2011). Although MTT assays may be influenced by redox-active compounds, they remain a standard initial screening method for cytotoxicity assessment. For adherent cells, the investigated compounds can be washed out before adding the substrate, in order to minimise the effect of redox reactions. Importantly, multiple studies have demonstrated that these catechins exhibit preferential toxicity toward cancer cells compared with normal fibroblasts. EGCG showed 4-fold higher toxicity to cancer cells (A549) than to normal fibroblasts (IC_50_: 25 μM vs. 100 μM) (Huang et al., 2017). Our results also confirm this correlation. EGCG notably reduced the number of viable A549 pulmonary tumour cells after 48 h (Figure 1D). A comparable outcome was seen in the MDA-MB-231 triple-negative breast cancer cell line, where both EGCG and EGC caused a significant decrease in viability after 48 h (Figure 1F). As reported in the literature, catechins exhibit concentration-dependent cytotoxic effects on MDA-MB-231 triple-negative breast cancer cells, with efficacy varying across different concentration ranges and exposure durations. Zeng et al. reported that the primary effect of EGCG therapy alone on MDA-MB-231 was the induction of cell death, as demonstrated in our study (Zeng et al., 2014).

Particular attention should be paid to the effects of extracts from *R. japonica* roots from *in vitro* culture (Figure 2). Analysis of the anticancer activity of the extracts showed that, at the concentration used, they reduce the viability of lymphoma cells (Ramos, Raji, and Namalwa) without affecting the viability of healthy skin fibroblasts, a promising effect. It should be noted that, when calculated based on the obtained HPLC results (Supplementary Table S1) at the highest concentration used (1,000 µg dw/mL), the extracts contained 26.84 μM EC, 10.82 µM EGC, 4.57 µM ECG, and 1.52 µM EGCG, while at the lowest concentration used (125 µg dw/mL) they contained 3.36 μM EC, 1.35 µM EGC, 0.57 µM ECG, and 0.19 µM EGCG. We therefore observed a similar effect to that obtained with pure reference compounds, except that their content in the extracts was significantly lower, which indicates the likelihood of synergistic or additive interactions when plant extracts are used. This observation indicates the advantage of using the extract rather than the pure test substances. Importantly, the anticancer effect on Ramos, Namalwa, and Raji cells is evident even at the lowest concentration tested. Despite the lack of prior reports on the effects of *R. japonica* extracts, other researchers generally report that multiple plant extracts demonstrate dose-dependent cytotoxicity in lymphoma cell lines, with significant antiproliferative effects observed across different compounds (Cho et al., 2020). Our research confirms these observations, creating promising prospects for deepening our understanding of the response of lymphoma cells to the action of naturally occurring secondary metabolites. However, regardless of the concentration used, root extracts do not exhibit cytotoxic or antiproliferative activity against A549 and MDA-MB-231 cells, likely due to the lower susceptibility of these cell lines to catechins (Figures 2B,C).

Moreover, to determine the mechanism underlying the reduction in cell viability, the effects of the extracts on the cell cycle and the type of cell death induced were examined. The results indicate a clear cell line-dependent response to catechins and the root extract, highlighting differences in cellular sensitivity to these compounds. The lack of detectable cell cycle alterations in BJ and A549 cells suggests a relative resistance to catechin-mediated effects, which is consistent with reports highlighting the selective antiproliferative activity of polyphenols across different cancer types. Interestingly, in A549 cells, a decrease in the MTT signal was observed without significant changes in the cell cycle (Figure 3B), as analysed by cytometry, suggesting a slowdown in proliferation without a classic cell cycle arrest. Catechins have been shown to modulate the expression of cell cycle regulators, for instance, through the upregulation of cyclin-dependent kinase inhibitors (CKIs), p21 and p27. While these suppressor proteins are typically downregulated in cancer cells to allow continuous division, catechin treatment restores their expression. This dose-dependent excess of p21 and p27 leads to the direct binding and inhibition of cyclin E1/CDK2 complexes. By preventing DNA replication, this mechanism effectively prolongs overall cell cycle duration and halts the expansion of cancer cells (Li et al., 2022; Sun et al., 2020). In addition, they can induce senescence, i.e., permanent inhibition of cell division while maintaining cell survival, thereby reducing metabolic activity as measured by the MTT assay. One of the adaptive mechanisms may also be cytoprotective autophagy (Sun et al., 2020). In contrast, significant cell cycle disturbances observed in MDA-MB-231 and all lymphoma cells (Ramos, Namalwa, Raji) indicate increased susceptibility to cell cycle interference (Figures 3C–F). The accumulation of cells in the S phase in MDA-MB-231 cells and in Ramos cells treated with ECG or EGC suggests replication stress or inhibition of DNA synthesis, potentially mediated by disruption of cyclin-dependent kinase activity. Conversely, the G2-phase arrest observed in EGCG-treated Ramos cells and in plant extract-treated Raji and Namalwa cells points to activation of the G2/M checkpoint, possibly in response to DNA damage (Luo et al., 2024; Li et al., 2022). Notably, the pronounced accumulation of Namalwa, Raji, and especially Ramos cells in the subG1 fraction following root extract treatment, together with reduced S- and G2-phase populations in Ramos, indicates strong antiproliferative and pro-apoptotic effects. This suggests that the extract exerts broader and more potent biological activity than individual catechins (despite its lower catechin content relative to the concentrations of the standards we used), likely due to synergistic or additive interactions or additional bioactive constituents. Extracts obtained from plants are also rich in phenolic acids such as ferulic acid, caftaric acid, chlorogenic acid, caffeic acid, and protocatechic acid (Makowski et al., 2024), which also exhibit anticarcinogenic properties (Sun and Shahrajabian, 2023). The sum of the above-mentioned phenolic acids in the plant extracts, calculated from the HPLC results (Supplementary Table S1), was 8.18 μM at a concentration of 1,000 µg dw/mL. In lymphoma cell lines, we also observed the emergence of a small fraction (from 1% to 3%) of polyploid cells, which was significantly elevated (up to 11%) in the Namalwa cell line following the application of plant extracts (Figures 3D–F). Two main models of this phenomenon can be proposed. In the first model, the catechins contained in the extracts may act as factors that disrupt microtubule organisation, inducing mitotic arrest followed by mitotic slippage, which directly leads to the formation of polyploid cells. In the second model, DNA damage and oxidative stress play a dominant role: they activate and subsequently destabilise cell cycle control mechanisms, thereby enabling DNA replication without cell division (endoreduplication) (Liu et al., 2024). These two mechanisms are not mutually exclusive and may co-occur, leading to the observed increase in the fraction of cells with elevated DNA content in the lymphoma cell lines studied.

The results show that the pro-apoptotic activity of the investigated substances is dependent on both the compound and the cell line (Figure 4). ECG caused a minor but statistically significant increase in apoptosis in MDA-MB-231 cells, whereas EGCG showed a similar, albeit non-significant, trend, suggesting that this cell line is less sensitive to catechin-induced apoptotic signalling (Figure 4C). In contrast, Ramos, Namalwa, and Raji cells exhibited a more pronounced apoptotic response, particularly after ECG therapy, indicating that this haematological cancer model is more susceptible to catechin-mediated apoptosis (Figures 4D–F). Similar trends have been found in other studies. Noda et al. demonstrated that EGCG induces apoptotic death in Ramos cells (Noda et al., 2007). Notably, the root extract provoked a pronounced apoptotic response in all studied lymphoma cell lines (Ramos, Namalwa, Raji), validating its previously reported antiproliferative properties and linking cell cycle disruption to apoptosis induction. The absence of necrotic cell death across all treatments suggests that the observed reduction in cell viability is primarily attributable to regulated, apoptosis-driven mechanisms rather than nonspecific cytotoxicity. Collectively, these findings imply that the tested compounds preferentially activate programmed cell death pathways in sensitive cancer cell types, with complex plant extracts exerting more potent effects than isolated catechins, potentially due to (previously mentioned) additive or synergistic interactions among bioactive constituents.

A toxicity test was then conducted using a standard insect model to confirm the safety of the extract from the roots of *R. japonica* and the reference compounds (Figure 5). Over the 120 h observation period, the control group’s larval survival remained high. Only slight decreases in survival were observed after treatment with the plant extract and the catechins (EGC, EGCG, EC, and ECG), mostly within the first 48 h of exposure. Crucially, no progressive or cumulative mortality was observed during the experiment, and survival rates stabilised thereafter. In addition, further steps were taken, and histograms of lymph collected from the larvae were analysed with a cell counter (data not shown); no changes were observed in lymph composition. Overall, these results suggest that the plant extract and individual chemicals are generally well tolerated by larvae and do not cause significant or long-term detrimental effects under the conditions investigated. Some of the available literature data indicate that phenolic plant extracts, in particular polyphenolic fractions, significantly reduce the survival rate of *G*. *mellonella* larvae in a manner dependent on dose, exposure time, and phytochemical composition, with extracts from *Aneth graveolens* exhibiting a stronger lethal effect than those from *Coriandrum sativum* (Oulebsir-Mohandkaci et al., 2018). Other studies show that plant extracts containing phenolic compounds are generally well-tolerated by larvae and in some cases can have immunomodulatory and antioxidant effects and enhance protective mechanisms of innate immunity (Das Chagas Almeida et al., 2019; Kaya et al., 2021; Battal et al., 2026). This indicates that the *G. mellonella* model is sufficiently sensitive to detect biologically relevant differences in the *in vivo* safety profile of plant-derived preparations. In contrast, we found no prior evidence in the literature on the effects of *R. japonica* extracts on larvae. At the same time, there is a lack of clear data in the literature on the direct effects of catechins and their derivatives on the survival of *G. mellonella* larvae, as previous studies have primarily relied on complex polyphenol mixtures (Das Chagas Almeida et al., 2019; Kaya et al., 2021; Battal et al., 2026; Oulebsir-Mohandkaci et al., 2018). Therefore, studies using catechins and their derivatives fill an important knowledge gap, enabling an unambiguous assessment of their effects on larval survival and a better understanding of the mechanisms of action of phenols in this insect model.

These results not only confirm the biological activity of the analysed compounds but also open up a promising path for further research into their potential anticancer activity in selected cell lines. Our study also, for the first time, describes the effect of extracts from *R. japonica* roots grown *in vitro* on both cancer cell lines and a simple insect model. In addition, the observations suggest the possibility of synergistic or additive interactions among the compounds in the extracts, clearly demonstrating the advantage of using *R. japonica* extracts over pure substances. This may also provide an important basis for further, more complex analyses of molecular mechanisms and for optimising therapies using natural bioactive compounds from *R. japonica*.

## Data Availability

The original contributions presented in the study are included in the article/Supplementary Material, further inquiries can be directed to the corresponding authors.
