# Supplementary material for: Anticancer potential of Reynoutria japonica Houtt. in vitro root extract on lymphoma cell lines
Source: Front Pharmacol. 2026 Aug 14;17:1904230. doi: 10.3389/fphar.2026.1904230 (PMC13522145; doi:10.3389/fphar.2026.1904230)
Supplement: Supplementary file 1 [file Table1.docx]

**Anticancer potential of *Reynoutria japonica* Houtt. *in vitro* root extract on lymphoma cell lines**

Julia Sroka^1^, Mikołaj Jacyno^2,3^, Dariusz Nowicki^4^, Wojciech Makowski^5^, Agnieszka Szopa^6^, Paweł Kubica^6^, Aleksandra Królicka^1^*, Małgorzata Stasiłojć^7^*

^1^Laboratory of Biologically Active Compounds, Intercollegiate Faculty of Biotechnology UG and MUG, University of Gdansk, Abrahama 58, 80-307 Gdansk, Poland

^2^Department of Immunology, Mossakowski Medical Research Institute, Polish Academy of Sciences, Adolfa Pawińskiego 5, 02-106, Warsaw, Poland

^3^Doctoral School of Translational Medicine, Mossakowski Medical Research Institute, Polish Academy of Sciences, Centre of Postgraduate Medical Education, Marymoncka 99/103, 01-813, Warsaw, Poland

^4^Department of Bacterial Molecular Genetics, Faculty of Biology, University of Gdansk, Wita Stwosza 59, 80-308 Gdansk, Poland

^5^Department of Botany, Physiology and Plant Protection, Faculty of Biotechnology and Horticulture, University of Agriculture in Kraków, 29 Listopada 54, 31-425 Kraków, Poland

^6^Department of Medicinal Plant and Mushroom Biotechnology, Jagiellonian University Medical College, Medyczna 9, 30-688, Kraków, Poland

^7^Department of Cell Biology and Immunology, Intercollegiate Faculty of Biotechnology of the University of Gdansk and Medical University of Gdansk, Debinki 1, 80-211, Gdansk, Poland

*Corresponding authors: [malgorzata.stasilojc@gumed.edu.pl](mailto:malgorzata.stasilojc@gumed.edu.pl), [aleksandra.krolicka@ug.edu.pl](mailto:aleksandra.krolicka@ug.edu.pl)

**Supplementary Table 1.** Accumulation of flavan-3-ols [mg × 100 g−1 DW] and phenolic acids [mg × 100 g−1 DW] in *R. japonica* anatomical roots cultivated *in* *vitro*, performed by useing DAD-HPLC. DW – dry weight, SD – standard deviation.

| Secondary metabolite | mg × 100 g DW^-1^ [± SD] | *R. japonica* roots  *in vitro* culture |
| --- | --- | --- |
| Catechin |  | 445.94 ± 12.93 |
| Epigallocatechin |  | 331.31 ± 34.59 |
| Epigallocatechin gallate |  | 69.58 ± 8.36 |
| Epicatechin |  | 779.18 ± 18.50 |
| Epicatechin gallate |  | 202.01 ± 15.66 |
| Catechin gallate |  | 341.52 ± 22.29 |
| Protocatechuic acid |  | 6.18 ± 0.37 |
| Coffee acid |  | 12.56 ± 1.42 |
| Feluric acid |  | 137.04 ± 19.04 |
|  |  |  |
